# Urethral Stricture Formation Following Cuff Erosion of AMS Artificial Urinary Sphincter Devices: Implication for a Less Invasive Explantation Approach

**DOI:** 10.3389/fsurg.2022.829517

**Published:** 2022-02-09

**Authors:** Katharina Kuhlencord, Roland Dahlem, Malte W. Vetterlein, Raisa S. Abrams-Pompe, Valentin Maurer, Christian P. Meyer, Silke Riechardt, Margit Fisch, Tim A. Ludwig, Phillip Marks

**Affiliations:** ^1^Department of Urology, University Medical Center Hamburg-Eppendorf, Hamburg, Germany; ^2^Department of Urology, Asklepios Medical Center Harburg, Hamburg, Germany; ^3^Department of Urology, Clinic Herfurt, University of Bochum, Herfurt, Germany

**Keywords:** complications, AMS 800™, artificial urinary sphincter, urethral erosion, reconstructive urology, stress urinary incontinence, urethral stricture

## Abstract

**Objectives:**

The objective of this study is to describe a standardized less invasive approach in patients with artificial urinary sphincter (AUS) explantation due to cuff erosion and analyze success and urethral stricture rates out of a prospective database. Evidence regarding complication management is sparse with heterogenous results revealing high risk of urethral stricture formation despite simultaneous urethroplasty in case of AUS explantation.

**Patients and Methods:**

Data of all patients undergoing AUS implantation due to stress urinary incontinence (SUI) in our tertiary center were prospectively collected from 2009 to 2015. In case of cuff erosion, AUS explantation was carried out in an institutional standardized strategy without urethroplasty, urethral preparation or mobilization nor urethrorrhaphy. Transurethral and suprapubic catheters were inserted for 3 weeks followed by radiography of the urethra. Further follow-up (FU) consisted of pad test, uroflowmetry, postvoiding residual urine (PVR), and radiography. Primary endpoint was urethral stricture rate.

**Results:**

Out of 235 patients after AUS implantation, 24 (10.2%) experienced cuff erosion with consecutive explantation and were available for analysis. Within a median FU of 18.7 months after AUS explantation, 2 patients (8.3%) developed a urethral stricture. The remaining 22 patients showed a median Qmax of 17 ml/s without suspicion of urethral stricture. Median time to reimplantation was 4 months (IQR 3-4).

**Conclusion:**

We observed a considerably low stricture formation and could not prove an indication for primary urethroplasty nor delay in salvage SUI treatment possibilities. Therefore, the presented standardized less invasive explantation strategy with consequent urinary diversion seems to be safe and effective and might be recommended in case of AUS cuff erosion.

## Introduction

Given the increasing uptake of treatment for benign or malignant prostatic diseases such as radical prostatectomy, radiotherapy, or endoscopic approaches, the incidence of SUI resulting from these procedures is rising despite improved surgical techniques (1). Consequently, this goes in hand with a profound negative impact on health-related quality of life and increased risk of institutionalization (1). SUI rates following radical prostatectomy range between 13% and 36% at one year after surgery (2, 3). Demographic changes with anticipated rising rates of abovementioned procedures reinforce the need for SUI management with long-term safety and efficacy to restore patients' quality of life. AUS implantation represents the gold-standard treatment for severe SUI with convincing early functional outcomes but high rates of troubling long-term complications requiring surgical revisions in up to 50% of cases (4, 5). Particularly, in almost one out of 10 patients (8.5%), AUS erosion with the subsequent need of AUS explantation represents a major complication as reported in a systematic review of 623 patients (6). Among others, hypertension, coronary heart disease, previous AUS erosion or infections, and prior RT have been identified as the risk factors for cuff erosion (7–11). Cuff erosion is known to unfavorably impact the likelihood of stricture formation compromising or impeding AUS reimplantation. Stricture rates following AUS erosion range from 8.3% up to 85% depending on the primary treatment protocol (12–14). Traditionally, the primary approach in case of erosion comprised AUS explantation and transurethral catheterization for urinary diversion (15, 16). However, emerging evidence suggests the feasibility of synchronous urethral repair at the time of AUS explantation to even reduce stricture development (14, 17). In a small series, a novel *in situ* urethroplasty (ISU) technique was superior over Foley catheter alone at the time of cuff erosion regarding both stricture development and the feasibility of secondary AUS implantation, although such results have to be interpreted carefully due to inherent selection bias (14).

Against this backdrop and given the ongoing debate regarding the optimal treatment of cuff erosions, we analyzed a large prospectively collected AUS database to evaluate stricture formation rates and report functional success in a homogeneously treated patient cohort requiring AUS explantation.

## Patients and Methods

### Study Population

This was an institutional review board-approved retrospective analysis of a prospective monocentric dataset including men undergoing AUS implantation for SUI (levels III and IV) from 2009 to 2015. Patient's informed consent was obtained and baseline patient characteristics, clinical variables, and follow-up were prospectively collected. Exclusion criteria for AUS implantation were mild SUI, eligibility for male sling implantation, an intact external sphincter, urodynamic evidence of detrusor overactivity during the first 300 ml of filling cystometry, a low bladder capacity <300 ml or urinary tract infection.

#### Perioperative Management—AUS Implantation

Perioperative management and AUS implantation were performed according to a standardized protocol by high-volume surgeons as previously described (18, 19). Preoperatively, patients underwent an examination by urodynamic cystometry, cystoscopy, pad test, and urinanalysis. An i.v. antibiotic regime (cefuroxime + gentamicin) was initiated 1 day prior to surgery and was continued for 5 days.

After placing the patient in a lithotomy position, a perineal incision was used to isolate the bulbar urethra to place the cuffs. Depending on the medical history of the patient, a distal bulbar or transcorporal or membranous cuff position was used. Cuff size was determined by loose measurement of urethral circumference to reduce permanent pressure to urethral tissue and resulting in a median cuff size of 4.5 cm.

After the procedure, the AUS is deactivated and a transurethral catheter (12 F) is inserted. On the third postoperative day, the transurethral catheter is removed and PVR is measured. Before discharge, positioning and filling of the AUS are controlled radiographically. A radiographic controlled activation of the AUS takes place 6 weeks after implantation. Under inpatient conditions, patients are trained in the use of the AUS and instructed to avoid perineal pressure and also to present immediately to urological care and our clinic for reevaluation in case of signs of AUS malfunction or infection.

A routine reassessment is performed by pad test, uroflowmetry, PVR, and clinical examination combined with a standardized nonvalidated questionnaire. These procedures are performed 6, 12, and 24 months after surgery and subsequently every 2 years.

#### Perioperative Management—AUS Explantation

In case of radiographic or urethroscopic evidence of cuff erosion, we perform a complete AUS explantation within 6 h. Simultaneously, i.v. antibiotic therapy is started (cefuroxime + gentamicin) and culture-specifically adapted (urine culture + intraoperative swabs). The institutional standardized surgical approach comprises perineal incision and lateral preparation along the connected AUS tubes with minimal mobilization of surrounding tissue. As soon as sufficient visualization is obtained, the cuff(s) are opened or dissected and removed. For the purpose of minimal iatrogenic trauma, we refrain from further tissue mobilization, ISU, or urethrorrhaphy following cuff removal. Neither the corpus spongiosum nor the urethra is sutured. Wound closure is performed in three layers: m.bulbospongiosus, subcutaneous, and intracutaneous sutures. The remaining system is removed by a small incision in the lower abdomen. Both suprapubic (10 F) and transurethral catheter (18 F) are inserted to ensure urinary drainage for 3 weeks until a radiographic examination is performed to evaluate extravasation or stricture disease. In the absence of extravasation, both catheters are removed and micturition is permitted; otherwise, a second control is scheduled. A standardized second examination is performed 3 months after AUS explantation to evaluate the feasibility of reimplantation within the same criteria as mentioned for primary implantation including cystoscopy or RUG. In case of a subsequent Qmax <10 ml/s or decrease of >25% compared to previous results, patients are again reevaluated *via* urethrography or cystoscopy to rule out urethral stricture.

Characteristics of patients suffering from AUS erosion are compared to those without AUS erosion in our database. Primary endpoint of this study was the assessment of urethral stricture rate after explantation of AUS due to erosion carried out in an institutional standardized less invasive treatment strategy.

### Statistical Analyses

Descriptive statistics relied on the comparison of means and proportions, using the chi-squared test for categorical and the *t*-test for continuous variables. All analyses were performed two-sided with a considered level of significance set at *p* < 0.05. Analyses were performed using SPPS® 20 (SPSS Inc., IBM Corp., Armonk, NY, USA) and R version 3.5.1 (The R foundation).

## Results

Data and clinical characteristics of 235 patients who underwent AUS implantation at our institution were available for final analysis. Overall, 17 patients (all without cuff erosion until then) were lost to follow-up (6.8%).

Clinical characteristics of all 235 AUS patients stratified according to AUS erosion are presented in Table 1. Median follow-up was 33.5 months for the erosion group and 24 months for the comparison group without erosion. A total of 24 out of 235 patients (10.2%) experienced AUS erosion requiring explantation. Median age of the patients with erosion was 71 years (IQR 67–75). Of these, 9 (37.5%) patients had a history of open surgery for SUI, either a male sling or a prior AUS implantation. Pelvic radiotherapy was reported in 14 (58.3%) cases, 5 (20.8%) patients were diabetics. Both groups only differed significantly regarding anticoagulation therapy applied to 18 patients (75%) with AUS erosion and 71 patients (33.6%) out of the comparison group (*p* = 0.01). A history of pelvic irradiation tended to be distributed significantly different with 14 (58.3%) patients with AUS erosion and 68 (32.2%) without (*p* = 0.07). There was no statistically significant difference between the erosion group and the comparison cohort with respect to age, comorbidities, surgeries prior to AUS, ASA classification, or operation time.

**Table 1:** Clinical characteristics among patients stratified according to the presence of AUS erosion after AUS implantation.

**Patients, *n* (%)**	**Erosion** ***n =* 24 (100)**	**Comparison group** ***n =* 211 (100)**	***p-*value**
Median age at surgery, years (IQR)	71 (65.75–75.25)	70.0 (65.0–73.5)	*p =* 0.72
Median ASA classification (IQR)	2 (2–3)	2 (2–3)	*p =* 0.69
**Comorbidities**, ***n*** **(%)**			
– Diabetes mellitus – Anticoagulant therapy	5 (20.1) 18 (75)	22 (10.4) 71 (33.6)	*p =* 0.19 *p =* 0.01
Pelvic radiation therapy, *n* (%)	14 (58.3)	68 (32.2)	*p =* 0.07
**Surgeries prior AMS implantation**, ***n*** **(%)**			
– Open surgical therapy for SUI	9 (37.5)	61 (28.9)	*p =* 0.675
Median AUS operation time, minutes (IQR)	58 (47.75-68.5)	58 (52-68)	*p =* 0.66

Clinical characteristics of 24 patients who developed AUS erosion are outlined in Table 2, stratified to the presence of stricture formation. Compared to all AUS patients, clinical characteristics showed comparable age at surgery and operation times at AUS implantation. After explantation in above-mentioned approach, only 2 (8.3 %) patients developed urethral stricture disease after 6 and 10 months, respectively. Median FU for patients without urethral stricture was 19 months. The median duration of catheterization after AUS explantation was 5 weeks (3–12). The indwelling catheters could be removed after the first radiographic control in 18 of 24 patients. In four patients, the catheters could be removed after the second and in two patients after the third control. Regarding the two patients suffering from stricture formation, the catheters were removed after the first and in the other case after the third radiographic control, respectively. Both patients with urethral stricture formation had a history of pelvic radiotherapy and also anticoagulant therapy. None of them were diabetics. One patient had open surgery for SUI prior to AUS implantation. Both patients revealed urethral strictures in radiography which was treated with a buccal mucosa graft urethroplasty before undergoing replacement of an AUS.

**Table 2:** Clinical characteristics among patients stratified to the presence of stricture formation after AUS explantation due to erosion.

**Patients, *n* (%)**	**No stricture** ***n =* 22 (100)**	**Stricture** ***n =* 2 (100)**
Median age at surgery, years (IQR)	71 (65.75–75.25)	72.5 (65–72.5)
Median ASA classification (IQR)	2 (2–3)	2.5 (2–2.5)
**Comorbidities**, ***n*** **(%)**		
– Diabetes mellitus – Anticoagulant therapy	5 (22.7) 14 (63.6)	0 2 (100)
Pelvic radiation therapy, *n* (%)	13 (59.1)	2 (100)
**Surgeries prior AMS implantation**, ***n*** **(%)**		
- Open surgical therapy for SUI	7 (31.8)	1 (50)
Median AUS operation time, minutes (IQR)	57 (47–65.75)	62.5 (55–62.5)
Time to AUS reimplantation, months (IQR)	4 (3–4)	6 (–)

The 22 patients without suspicion for urethral stricture disease showed a median Qmax of 17 ml/s (IQR 15-27 ml/s) and no PVR after AUS explantation.

Altogether 22 patients (92%) met above-mentioned criteria and were eligible for a reimplantation of an AUS after a median time of 4 months (IQR 3–4 months).

## Discussion

Since growing numbers of prostate surgery entail a rising prevalence of male SUI, the implantation of AUS maintains a safe and effective treatment of great importance for patients and surgeons (6, 9, 20). Scientific evidence mainly addresses indication, surgical procedure, and device specifications only for AUS implantation. Therefore, considerably more evidence regarding complication management is required. In particular, urethral erosion after AUS implantation is a challenging problem resulting in AUS explantation subsequently leading to re-SUI with profound negative impact on patients' quality of life (13). Furthermore, AUS explantation is reported to come along with the high risk of urethral stricture formation and subsequent interventions delay a possible salvage treatment of the SUI.

This stresses the need for a safe and effective treatment strategy of AUS explantation with minimal rates of stricture formation, and therefore, fewer following interventions offer the possibility of an early AUS replacement.

In recent years, the discussion focused on the choice of surgical approach during AUS removal due to urethral erosion and the necessity of a concomitant urethroplasty.

Literature evaluating the management of cuff erosions aiming to avoid stricture formation is scarce. To the best of our knowledge, only few retrospective studies with inhomogeneous treated patient cohorts are available, data are conflicting, and level of evidence is low.

In this study, we analyzed a prospectively collected database regarding outcomes of an institutional standardized less invasive treatment approach in case of AUS erosion without primary urethral repair, and the following findings are worthy of discussion.

First, to put our results into context, we summarized outcome studies after AUS explantation in Table 3. In detail, a retrospective multicenter analysis comprising outcomes of 78 patients after AUS explantation by Gross et al. stated the lowest stricture formation after AUS explantation with concomitant urethroplasty. When comparing either urethrorrhaphy (urethral stricture rate 40%), urethroplasty (14%), or catheter drainage alone (29%), these differences did not reach statistical significance (13). Rozanski et al. retrospectively compared 26 patients undergoing AUS explantation with or without concomitant *in situ* urethroplasty (ISU) with equal numbers of 13 patients in each group (14). The rate of urethral stricture formation was also described as significantly lower after treatment with simultaneous ISU affecting 5 patients in contrast to 11 patients after single Foley catheter urinary drainage. However, these data show overall stricture rates of 38% vs. 87% for these treatment groups (14). Another comparative analysis did not reveal significant differences in stricture formation after AUS explantation with Foley catheter placement (17%) or abbreviated urethroplasty (33%) or mobilization with primary anastomosis (25%) (21). Although a majority of authors suggest concomitant urethroplasty in case of urethral erosion in AUS patients, we could show considerably lower stricture rates carrying out a standardized, less invasive treatment approach (13, 14, 21–23). Overall, the existent literature shows inhomogeneous results and data depict rates of urethral stricture formation in 14–87% of all patients and 17–87% after Foley catheter treatment alone. In contrast, our results reveal a lower stricture formation rate of 8.3% after AUS explantation. Hence, this represents the largest cohort homogenously treated by a standardized approach. However, our results corroborate another previously published series by Agarwal et al. from the Mayo Clinic, who analyzed the management and outcomes of AUS explantation in 63 patients between 1983 and 2011 (12). Their treatment strategy comprised AUS explantation with no concomitant urethroplasty and indwelling catheter for 4–6 weeks in 58 (92%) patients. In four patients, simultaneous urethroplasty at the time of AUS explantation was performed, and one patient underwent urethral ligation with suprapubic catheterization. Supporting our findings of a prospectively collected institutional database, the risk of stricture formation was reported 8.3% in patients after AUS explantation without concomitant urethral reconstruction (12). However, examination by retrograde urethrography was available only in 36 (62.1%) patients in this cohort whereas standardized follow-up of all our patients comprises results of urethrography.

**Table 3:** Summary of studies reporting management and stricture rates of AUS explantation in case of urethral erosion.

**Study**	**Urethral stricture rate (type of repair/no pat./%)**	**Protocol/aftercare**	**Imaging or cystoscopy (UC)**	**Number of patients/** **type of treatment**		**Duration of urinary diversion after AUS expl**.		**Follow-up after AUS expl**.	**Study design/randomization**
				**AUS explantation + UP/PA/UR**	**AUS explantation + urinary diversion**	**Foley catheter**	**Suprapubic catheter**		
Agarwal et al. (12)	Foley only: 3 (8.3%)	Pericatheter RUG 6 weeks after AUS removal	pericatheter RUG or UC	4 ISU 1 urethral ligation	58	4–6 weeks	1 (urethral ligation)	n.s.	Retrospective/ single center/no
Chertack et al. (21)	AU: 1 (33 %) PA: 2 (25%) Foley only: 6 (17%)	Foley 3–6 weeks	VCUG	8 AU 15 PA	52	3–6 weeks	excluded from analysis	21 months, mean	Retrospective/ single center/no
Gross et al. (13)	UR: 17 (40%) UP: 2 (14 %) Foley: 6 (29%)	n.s.	RUG or UC	43 UR 14 ISU	21	n.s.	n.s.	n.s.	Retrospective/ multi-institutional/no
Rozanski et al. (14)	UP: 5 (38%) Foley only: 11 (85%)	Foley 3 weeks	VCUG 3 weeks postoperatively UC two months after VCUG	13	13	3 weeks	n.s.	24 months, mean	Retrospective/ single center/no

Overall, these studies and differing results lead to the assumption that varying surgical approaches and catheter drainage strategies throughout different surgeons or institutions possibly affect results after AUS explantation and their comparability.

Particularly, our treatment strategy comprises sufficient urinary drainage with suprapubic plus transurethral catheter insertion whereas other studies describe transurethral catheterization only (13, 14, 21). This combined urinary drainage is related to principles of conservative and surgical management of urethral trauma and fistula and may represent one main difference to previously described AUS explantation approaches. Further to date, no standardized recommendation of antibiotic therapy exists and its use is not reported in most studies. Therefore, we propose an immediate broad i.v. antibiotic regime consisting of cefuroxime and gentamicin and conversion regarding microbiological results of intraoperative swab test which might further influence results in case of erosions.

Moreover, the consideration to implement a less invasive AUS explantation approach was based on clinical experience and the idea of avoiding further extent of tissue damage and interference of microcirculation with a risk of enhanced scar formation due to ischemic fibrosis (24). Due to the well-known adverse impact of urethral tissue impairment on urethral recovery and reconstruction, we recommend urethroplasty only in case of stricture formation in course of the treatment follow-up. With respect to a low stricture rate of 8.3% after AUS explantation without ISU, the majority of patients did not need further reconstructive surgery.

In this regard, Gross et al. notably revealed that the risk of urethral stricture formation after AUS explantation is significantly driven by the extent of urethral erosion (13). A similar correlation is demonstrated by Chertack et al. which shows significantly higher rates of urethral stricture formation after AUS explantation and Foley catheter placement in patients with erosions comprising >50% of urethral circumference. Besides these two studies reporting different degrees of urethral erosion, Rozanski et al. describe repairs of entirely urethral defects in their cohort (14). This might be of importance concerning the further explanation of conflicting results and underlines difficulties to compare data of previous AUS explantation outcome studies. As above-mentioned, we avoid preparation of the eroded urethra and further exploration of the defect. Thus, and in contrast to other studies, the explantation approach did not change with various extent of urethral erosion in our study.

Interestingly and in line with our institutional standards, Agarwal et al. reported not to use cuff size <4.0 cm for AUS implantation (12). Thereby, this is true for both studies showing the lowest stricture rates in case of AUS erosion. The impact of AUS cuff size on outcomes and complication rates is still discussed (25), though most authors did not report increased adverse results for the implantation of smaller AUS cuff sizes (26, 27). Regarding urethral erosions, our findings together with Agarwal et al. might point to a possible influence on urethral erosions and following risk of urethral stricture formation, which needs further investigation.

Concerning above-mentioned difficulties to compare previous AUS explantation studies, previous retrospective analyses overall show considerably differing caseloads in each treatment group. Furthermore, within description of varying surgical approaches, studies lack details of decision-making or randomization regarding treatment approaches in individual patients. Thus, we describe a standardized treatment strategy carried out in all AUS explantations from 2009 onward, resulting in a homogenous patient cohort.

In terms of subsequent treatment patterns for recurrent urinary incontinence after AUS explantation, Rozanski et al. stated a considerable delay of AUS reimplantation for patients without ISU (17 months) compared to patients after explantation and simultaneous ISU (9 months) (14). Related to our institutional approach, indwelling suprapubic and transurethral catheter is removed after unsuspicious RUG not earlier than 3 weeks postoperatively. After a period of further 3 months without suspicion of urethral stricture formation, finally controlled by RUG, a salvage AUS implantation is evaluated. Considering this clinical pathway, AUS reimplantation was performed 4–6 months after explantation. In accordance with some other authors, a less invasive AUS explantation strategy does thereby not account for delay or diminished probability of further treatments in patients with AUS erosion despite individual courses of disease (21). In case of stricture formation, required treatment is initiated from that point onward. Despite a low rate of stricture formation after AUS explantation, we could previously show successful AUS reimplantation taking place 3 months after buccal mucosa graft urethroplasty (28).

## Limitations

To our knowledge, this study represents the first study of AUS explantation outcomes from a prospectively collected database. However, we acknowledge some limitations. Although data were collected in a prospective database, our analysis was performed retrospectively *post-hoc*. The sample size of AUS explantations and even more stricture formation make comparisons more difficult. Since the extent of urethral erosions seems to impair outcomes and risk of stricture formation, our data do not comprise a specific graduation of urethral erosion.

## Conclusion

The described institutional standardized less invasive AUS explantation with consequent combined urinary diversion represents a safe and effective approach in case of urethral erosions. The risk of stricture formation was considerably lower compared to published techniques and results. Furthermore, a described delay of possible AUS reimplantation was not evident.

A prospective randomized study could increase the level of evidence when comparing treatment strategies in case of AUS complications.

## Data Availability Statement

The raw data supporting the conclusions of this article will be made available by the authors, without undue reservation.

## Ethics Statement

Ethical review and approval was not required for the study on human participants, in accordance with the local legislation and institutional requirements. Written informed consent from the patients/participants was not required to participate in this study in accordance with the national legislation and the institutional requirements.

## Author Contributions

KK and RD: project development, data collection, data analysis, and manuscript writing. MV: manuscript editing and data analysis. RA-P, VM, CM, and SR: manuscript editing. MF: project development and manuscript editing. TL: data collection and data analysis. PM: project development, data analysis, and manuscript writing. All authors contributed to the article and approved the submitted version.

## Conflict of Interest

MF and RD worked as a consultant for Boston scientific. The remaining authors declare that the research was conducted in the absence of any commercial or financial relationships that could be construed as a potential conflict of interest.

## Publisher's Note

All claims expressed in this article are solely those of the authors and do not necessarily represent those of their affiliated organizations, or those of the publisher, the editors and the reviewers. Any product that may be evaluated in this article, or claim that may be made by its manufacturer, is not guaranteed or endorsed by the publisher.

## Abbreviations

ASA: American society of Anesthesiologists
AU: abbreviated urethroplasty
AUS: artificial urinary sphincter
FU: follow-up
IQR: interquartile range
PA: primary anastomosis
PVR: postvoid residual volume
SUI: stress urinary incontinence.
